# Downregulation of Splicing Factor *PTBP1* Curtails *FBXO5* Expression to Promote Cellular Senescence in Lung Adenocarcinoma

**DOI:** 10.3390/cimb46070458

**Published:** 2024-07-19

**Authors:** Haoyu Li, Xiaoxiao Sun, Yuanyuan Lv, Gang Wei, Ting Ni, Wenxin Qin, Haojie Jin, Qi Jia

**Affiliations:** 1 State Key Laboratory of Systems Medicine for Cancer, Shanghai Cancer Institute, Renji Hospital, Shanghai Jiao Tong University School of Medicine, Shanghai 200032, China; lihaoyu196@163.com (H.L.); smily5182@163.com (X.S.); ecnulyy66@163.com (Y.L.); wxqin@sjtu.edu.cn (W.Q.); hjjin1986@126.com (H.J.); 2 State Key Laboratory of Genetic Engineering, Collaborative Innovation Center of Genetics and Development, Human Phenome Institute, School of Life Sciences, Fudan University, Shanghai 200438, China; gwei@fudan.edu.cn (G.W.); tingni@fudan.edu.cn (T.N.)

**Keywords:** *PTBP1*, LUAD, alternative splicing, cellular senescence, *FBXO5*

## Abstract

Polypyrimidine tract-binding protein 1 (*PTBP1*) plays an essential role in splicing and post-transcriptional regulation. Moreover, *PTBP1* has been implicated as a causal factor in tumorigenesis. However, the involvement of *PTBP1* in cellular senescence, a key biological process in aging and cancer suppression, remains to be clarified. Here, it is shown that *PTBP1* is associated with the facilitation of tumor growth and the prognosis in lung adenocarcinoma (LUAD). *PTBP1* exhibited significantly increased expression in various cancer types including LUAD and showed consistently decreased expression in multiple cellular senescence models. Suppression of *PTBP1* induced cellular senescence in LUAD cells. In terms of molecular mechanisms, the silencing of *PTBP1* enhanced the skipping of exon 3 in F-box protein 5 (*FBXO5*), resulting in the generation of a less stable RNA splice variant, FBXO5-S, which subsequently reduces the overall *FBXO5* expression. Additionally, downregulation of *FBXO5* was found to induce senescence in LUAD. Collectively, these findings illustrate that *PTBP1* possesses an oncogenic function in LUAD through inhibiting senescence, and that targeting aberrant splicing mediated by *PTBP1* has therapeutic potential in cancer treatment.

## 1. Introduction

Lung cancer exhibits the highest global incidence and mortality rates among cancers, with lung adenocarcinoma (LUAD) being the most common histological type [1]. Despite significant advancements in therapeutic techniques and treatment modalities, challenges associated with the treatment of LUAD remain. Therefore, exploration of the underlying mechanisms is urgently needed to identify potential targets and establish theoretical foundations for drug design and clinical decision-making.

Cellular senescence is defined as a state in which cells exhibit an irreversible growth arrest due to various stressors such as DNA damage, oxidative stress, or telomere erosion, while still retaining metabolic activity that can modulate their microenvironment. Cellular senescence has a broad impact on maintaining tissue homeostasis and disease prevention, including neurodegenerative diseases, chronic inflammation, and tumor progression [2,3,4]. Typically, serving as an anti-tumorigenic barrier, cellular senescence inhibits the abnormal growth of cells that have sustained damage [5]. Senescent cells are characterized by several features, including increased activity of senescence-associated β-galactosidase (SA-β-Gal) [6] and the ability to release a wide range of bioactive compounds, known as the Senescence-Associated Secretory Phenotype (SASP), encompassing numerous signaling molecules [7]. In addition to changes in their chemical production, senescent cells experience morphogenesis as well. Generally, senescent cells are enlarged, flattened, and occasionally multinucleated [8]. The underlying mechanism of cellular senescence is widely believed to involve the p16-RB and p53-p21 tumor suppressor pathways [9]. These pathways play a crucial role in maintaining cellular homeostasis and in the prevention of tumorigenesis. p16^INK4a^ is a tumor suppressor protein that plays a crucial role in cell cycle regulation. As organisms age, the expression of p16^INK4a^ often increases, leading to a higher incidence of senescent cells. Therefore, p16^INK4a^ is considered a biomarker of cellular aging and is associated with the aging process at the cellular level [10]. In the p16-RB pathway, p16^INK4a^ inhibits the activity of cyclin-dependent kinases 4 and 6 (CDK4/6), preventing the cyclin D-dependent phosphorylation of the retinoblastoma protein (Rb), thereby maintaining Rb in its active, hypophosphorylated state. This prevents the cell cycle from progressing from the G1 to the S phase [11]. In the p53-p21 pathway, p53 is activated in response to DNA damage and upregulates its downstream target gene p21, which inhibits the activity of CDKs, leading to the activation of Rb and cell cycle arrest, thereby inducing cellular senescence [12]. However, there are many other mechanisms related to cellular senescence that require further investigation.

Alternative splicing (AS) is a regulated molecular mechanism that enables a single gene to produce a variety of mRNAs and proteins with potentially different cellular functions or properties. Approximately 95% of human genes undergo alternative splicing, which significantly expands the diversity within the transcriptome and proteome [13]. Dysfunctional alternative splicing leads to the emergence of various splicing variants and contributes to the regulation of a host of biological processes, including cell and tissue homeostasis and organ development [14,15,16,17,18]. Notably, in addition to transcriptional and epigenetic alterations, substantial alterations in alternative splicing are commonly observed during cellular senescence and tissue aging. Particularly, it has been suggested that dysregulation of splicing, due to changes in splicing factor expression, may contribute to the mechanisms underlying aging and senescence [19,20,21,22]. However, the exact mechanisms by which specific splicing factors influence senescence-related alternative splicing events and the ways in which their dysregulation may lead to cellular senescence have yet to be fully determined.

Polypyrimidine tract-binding protein 1 (*PTBP1*) plays a critical role in post-transcriptional gene regulation, affecting mRNA splicing, translation, stability, and cellular distribution [23,24,25]. *PTBP1* has a diverse range of molecular functions in RNA metabolism and acts as a dominant negative regulator of alternative splicing, leading to exon skipping in various pre-mRNAs [26]. Interestingly, a recent study indicated that the direct knockdown of *PTBP1* leads to particular attenuation of pro-inflammatory SASP factors in a variety of cell lines in vitro and in vivo, but functionally preserved oncogene-induced or therapy-induced senescence [23]. However, currently, the association between *PTBP1* and cellular senescence is still unclear and warrants further investigation. Given the link between senescence and cancer, and given that insights into how *PTBP1*-related alternative splicing events modulate senescence in LUAD are currently limited, here, we consider whether the regulation of *PTBP1* could influence senescence in LUAD patients.

In this study, we aimed to investigate the alternative splicing profile of *PTBP1* in LUAD and assess its impact on cellular senescence progression, along with its downstream effectors. We focused on one of the top misspliced targets upon *PTBP1* knockdown, *FBXO5*, a key regulator of cell cycle progression. Previous reports have mentioned two isoforms of *FBXO5* [27], but the mechanisms behind their generation and their roles in different cancers remain to be explored, as does the regulatory effect of splicing factors on *FBXO5*. Here, we demonstrate that *PTBP1* regulates the alternative splicing and expression of *FBXO5*, and that *PTBP1* depletion leads to exon 3 skipping of *FBXO5*, which, in turn, downregulates *FBXO5* and triggers cellular senescence in LUAD. Our study thus suggests that *PTBP1* may act as a potential novel therapeutic target for senescence-mediated tumor suppression.

## 2. Materials and Methods

### 2.1. Bioinformatics Database Sources

RNA-Seq profiles from the Tumor Immune Estimation Resource (TIMER) (https://cistrome.shinyapps.io/timer/ (accessed on 2 January 2024)) were utilized to assess *PTBP1* expression in 33 cancer types. The RNA-seq data of 542 LUAD samples and 59 normal samples were retrieved from the Gene Expression Profiling Interactive Analysis (GEPIA) (http://gepia.cancer-pku.cn (accessed on 22 March 2024)) for an in-depth exploration of gene characteristics and prognostic significance in LUAD. Additionally, the replicative senescence models (GSE63577) obtained from the GEO database (https://www.ncbi.nlm.nih.gov/geo (accessed on 10 September 2023)) provided insights into the mRNA profile of *PTBP1* during senescence.

### 2.2. Cell Culture and Transfection

The HEK293T cell line was cultured in Dulbecco’s modified Eagle’s medium (DMEM) supplemented with 10% fetal bovine serum (FBS). A549 and H1299 cell lines were cultivated in RPMI-1640 medium supplemented with 10% FBS. All cultures were incubated at a standardized 37 °C and in a 5% CO_2_ atmosphere. Cells were seeded onto 6-well or 12-well plates at 70–80% confluency for subsequent experiments. Stable knockdown of *PTBP1* and *FBXO5* in these cell lines was performed using lentiviral short hairpin RNA (shRNA). The sequences of shRNA are shown in Table 1. pLkO.1 was used as the control plasmid. Lentiviral particles were generated by co-transfecting HEK293T cells at 80% confluence in a 10 cm dish with a lentiviral vector (20 μg, pLVX: PxpAx2: PMD2g = 4:3:1:) at 37 °C for 48 h using Lip2000 (Invitrogen, Waltham, MA, USA). A549 and H1299 cells were infected with lentiviral particles, followed by drug selection (2 μg/mL puromycin for 3–5 days).

### 2.3. RNA Isolation and Quantitative Real-Time PCR Analysis

Total RNA was extracted from A549 and H1299 cell lines using an EZ-press RNA Purification Kit (EZBioscience, Roseville, MN, USA). Subsequent cDNA synthesis was performed with a Color Reverse Transcription Kit (EZBioscience, USA). Quantitative Real-Time PCR (RT-qPCR) was conducted on a LightCycler 480 Real-Time PCR System (Roche Applied Science, Indianapolis, IN, USA) using SYBR qPCR Master Mix (EZBioscience, USA), in accordance with the manufacturer’s instructions. The mRNA levels of target genes were normalized to the GAPDH gene. Reverse transcription-PCR (RT-PCR) was used to evaluate the mRNA levels of transcripts of target genes using 2 × Taq Master Mix (Dye Plus) (Vazyme, Nanjing, China). Primer information for RT-qPCR and RT-PCR is provided in Table 2.

### 2.4. Western Blot Analysis

Cells were lysed in RIPA buffer containing phosphatase and protease inhibitors. Protein quantification was conducted using a BCA Protein Assay Kit (P0012, Beyotime Biotechnology, Shanghai, China). Quantified cell lysates were separated by 10–15% SDS-PAGE and transferred to PVDF membrane, and they were then blocked with 5% BSA. Overnight incubation with primary antibodies was conducted at 4 °C, employing rabbit anti-PTBP1 (1:1000, D225103-0025, Sangon Biotech, Shanghai, China), rabbit anti-FBXO5 (1:1000, 10872-1-AP, Proteintech, San Diego, CA, USA), and anti-GAPDH (1:1000, HRP-60004, Proteintech). After incubation with HRP-linked secondary antibodies, protein detection was performed using enhanced chemiluminescence (Bio-Rad, Hercules, CA, USA). 

### 2.5. SA-β-Gal Staining

According to the manufacturer’s protocol of a senescence-associated β-galactosidase kit (C0602, Beyotime Biotechnology, Shanghai, China), cells seeded in 12-well plates were washed twice with PBS and fixed in 4% paraformaldehyde for 15 min. Cells were then incubated overnight with the working solution of β-galactosidase plus X-Gal at 37 °C followed by three washes with PBS.

### 2.6. Cell Proliferation and Colony Formation Assay

The cell proliferation rate was assayed using a Cell Counting Kit-8 (CCK-8) (Dojindo, Kumamoto, Japan). Briefly, cells were transplanted into 96-well plates with 100 μL culture medium and at least 2000 cells per well at 37 °C for 72 h. Then, 10 μL of CCK-8 solution was added to each well and incubated at 37 °C for 2 h, and the OD value of each well was determined by a microplate reader (BioTEK, Winooski, VT, USA). This measurement was performed every 24 h and a cell growth curve was drawn according to the absorbance value at each time point. For each well in a 6-well plate, 2 mL complete medium containing 200 cells was prepared. After culturing cells at 37 °C with 5% CO_2_ for 14 days, the supernatant was discarded and the plate was washed three times with PBS. Crystal violet (0.1%, Solarbio) was used to stain colonies, and the number of colonies was counted by Image J (version 2.3.0).

### 2.7. Cell Migration Assay

Cell migration was assessed using a 24-well transwell plate with an 8 μm pore size (Corning Life Sciences, Corning, NY, USA), in accordance with the manufacturer’s protocol. The upper compartment of the transwell filter was seeded with cells at a density of 5 × 10^4^ cells per well, using a medium that was fortified with 1% FBS. Medium containing 10% FBS was added to the lower plate. Following incubation for 24 h, the cells that had traversed to the lower filter were fixed in 4% paraformaldehyde, washed with PBS, stained using 1% crystal violet, and enumerated microscopically. Each experiment was repeated three times.

### 2.8. Cell Cycle Flow Cytometric Analysis

Cells were harvested and fixed in 70% ethanol at 4 °C overnight. According to the manufacturer’s protocol (BD pharmingen, San Diego, CA, USA), these fixed cells were incubated with PI/RNase Staining Buffer for 30 min at 37 °C in the dark after washing with PBS. The cellular DNA content was then quantified by flow cytometry (BD Biosciences, Milpitas, CA, USA). Analysis of the cell cycle distribution was conducted using ModFit 3.0.

### 2.9. mRNA Stability Assay

Cells were treated with 10 μg/mL Actinomycin D (ActD) for 0, 2, 4, 6, and 8 h, then harvested at each respective time point. RNA was extracted from these samples and subsequently reverse-transcribed into cDNA. The mRNA levels of two splice isoforms, FBXO5-L and FBXO5-S, were quantified using RT-qPCR. Primer information is provided in Table 2.

### 2.10. RNA-seq and Bioinformatics Analysis Methods

*PTBP1* knockdown A549 cells were applied followed by RNA sequencing. RNA-seq libraries for transcriptome-wide analysis were constructed according to the established methods [28,29]. RNA-seq libraries were sequenced using an Illumina HiSeq platform (San Diego, CA, USA). STAR [30] was used for mapping RNA-seq reads to human genome GRCh37 (https://github.com/alexdobin/STAR, accessed on 2 June 2024), and StringTie [31] was used for transcript assembly and quantification. rMATS [32] was used to analyze changes in alternative splicing including skipped exon (SE), mutually exclusive exon (MXE), alternative 3′ splice sites (A3SS), alternative 5′ splice site (A5SS), and intron retention (IR) in *PTBP1* knockdown A549 cells. The DESeq2 package (Version 1.26.0) was employed to identify the differentially expressed genes (DEGs) between *PTBP1*-high and *PTBP1*-low patients from TCGA datasets. The cut-off threshold was a |log fold change (FC)| ≥ 0.5 and adjusted *p*-value < 0.05. Pathway enrichment analysis in this study was performed using the Database for Annotation, Visualization, and Integrated Discovery (DAVID) [33].

### 2.11. Statistical Analysis

Experimental data were analyzed using GraphPad Prism 8.3.0 software. Results are presented as the mean ± SD (n = 3). Differences between two groups were evaluated using an unpaired *t*-test. A *p*-value < 0.05 indicated statistical significance. * represents *p* < 0.05; ** represents *p* < 0.01; *** represents *p* < 0.001.

## 3. Results

### 3.1. Opposite Levels of PTBP1 in LUAD and Cellular Senescence

**Figure 1:**
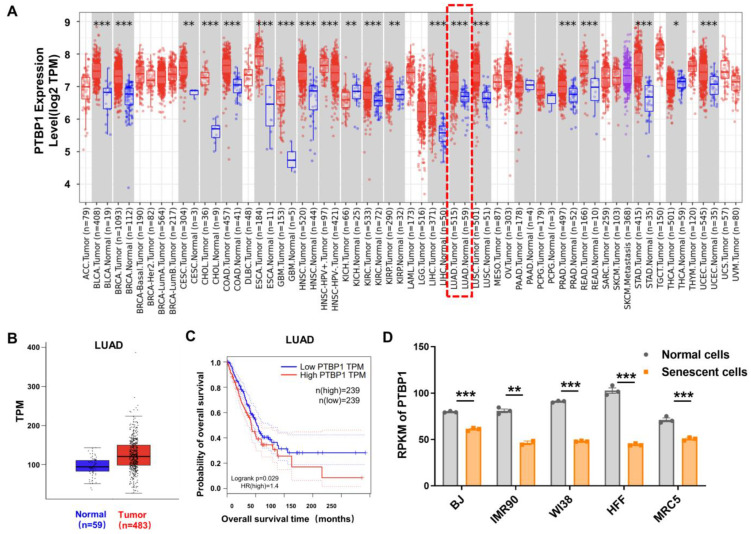
Expression levels of *PTBP1* in LUAD and senescent cells. (**A**) The expression of *PTBP1* among normal and tumor tissues including LUAD from TIMER. The expression of *PTBP1* in LUAD is marked by a red box. The red dots and blue dots within the rectangle represent tumor tissues and normal tissues, respectively, while the purple dots represent metastatic tissues. (**B**) Changes in mRNA levels of *PTBP1* in LUAD and matched normal tissues derived from GEPIA [34]. (**C**) Results from GEPIA showed that higher mRNA levels of *PTBP1* were associated with lower survival rates in LUAD patients. The solid line depicts the survival curve, and the dotted line represents the 95% confidence interval. A Log-rank test with a *p*-value < 0.05 is considered to indicate a statistically significant difference. (**D**) Decreased mRNA levels of *PTBP1* in five human senescent models compared to younger ones (* *p* < 0.05, ** *p* < 0.01, *** *p* < 0.001, *t*-test).

To systematically elucidate the expression pattern of the splicing factor *PTBP1* in cancers, we first analyzed the expression of *PTBP1* in 33 types of cancers based on the TIMER database. The results showed that the level of *PTBP1* was significantly increased in distinct tumors including LUAD (Figure 1A), suggesting that *PTBP1* may play an oncogenic role in tumor development. Further comparative analysis using the GEPIA database revealed a pronounced upregulation of *PTBP1* in samples of LUAD compared to non-cancerous pulmonary tissue, and higher *PTBP1* levels were also associated with lower survival rates of LUAD patients (Figure 1B,C).

Given the well-established significance of cellular senescence in cancer pathogenesis, we analyzed five replicative senescent human cell lines (BJ, IMR90, WI38, HFF, and MRC5) from the GSE63577 dataset. The senescent cells exhibited a discernible reduction in *PTBP1* expression (Figure 1D). Taken together, these data showed that *PTBP1* had an opposite expression trend in LUAD and cellular senescence, implying that *PTBP1* may affect the pathogenesis of LUAD by modulating cellular senescence.

### 3.2. Downregulation of PTBP1 Induces Senescence in LUAD Cells

**Figure 2:**
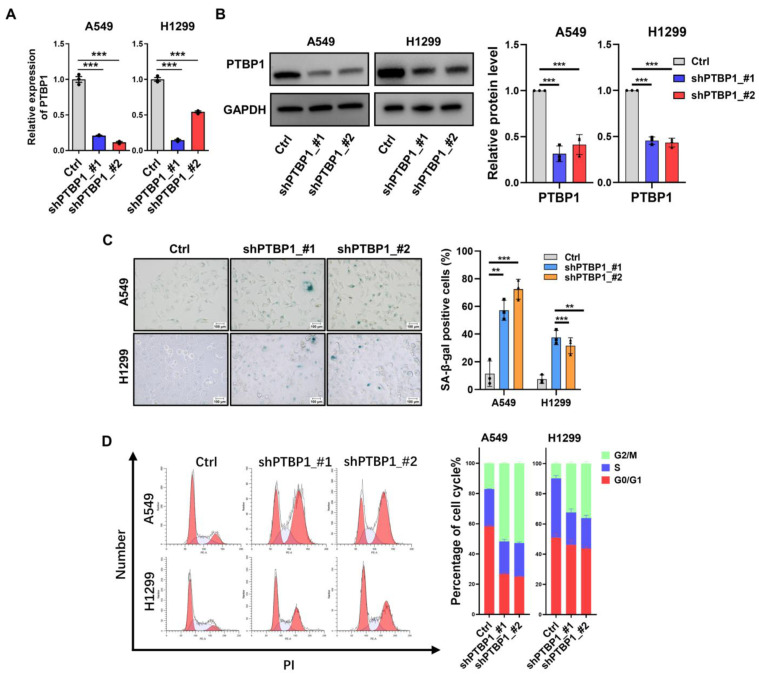
Knockdown of *PTBP1* induces senescence and cell cycle arrest in LUAD cells. *PTBP1* expression was validated by RT-qPCR (**A**) and Western blotting (**B**) in A549 and H1299 cells transfected with two different shRNAs (shPTBP1_#1 and shPTBP1_#2), with GAPDH serving as the internal control for both mRNA and protein levels. (**C**) SA-β-Gal staining in A549 and H1299 cells with *PTBP1* knockdown. (**D**) Cell cycle detection for A549 and H1299 cells with *PTBP1* knockdown. In the graph on the left side, red represents cells in the G1 and G2 phases. (** *p* < 0.01, *** *p* < 0.001, *t*-test).

**Figure 3:**
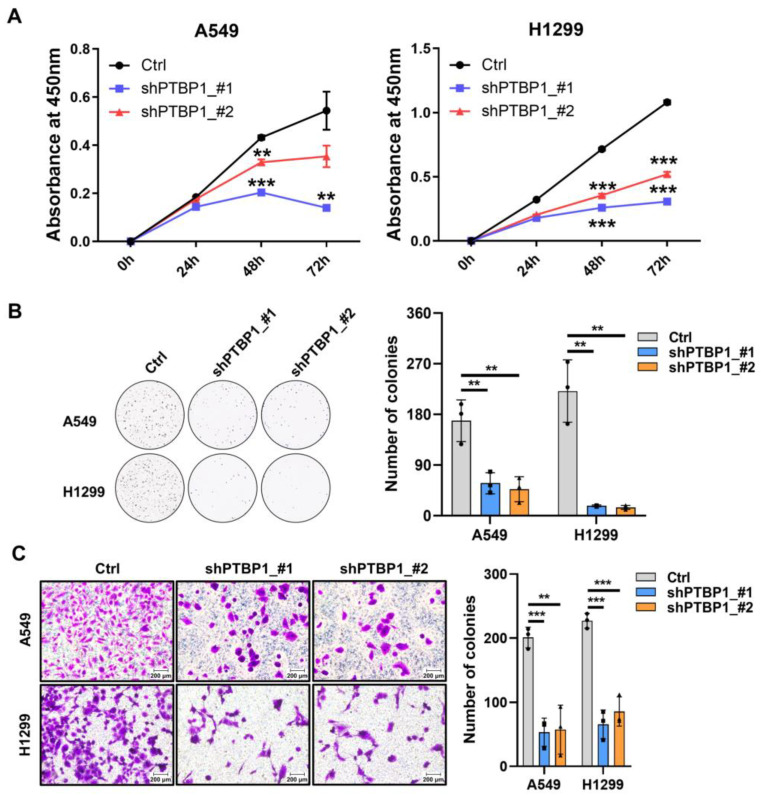
*PTBP1* knockdown inhibits the proliferation and migration of LUAD cells. (**A**) Cell proliferation rate of A549 and H1299 cells with *PTBP1* knockdown. (**B**) Colony formation capacity of A549 and H1299 cells with *PTBP1* knockdown. (**C**) Cell migration ability in A549 and H1299 cells with *PTBP1* knockdown (** *p* < 0.01, *** *p* < 0.001, *t*-test).

To investigate the role of *PTBP1* in LUAD and senescence, we implemented a loss-of-function strategy by transfecting A549 and H1299 cell lines with two targeted shRNAs against *PTBP1* (shPTBP1_#1 and shPTBP1_#2). The reduction in *PTBP1* mRNA and protein levels within these cancer cell lines was demonstrated and confirmed by RT-qPCR and Western blot analyses (Figure 2A,B). Further phenotypic analyses revealed that *PTBP1* knockdown (*PTBP1*-KD) cells displayed diminished proliferative and migratory properties (Figure 3A–C). As anticipated, SA-β-Gal staining increased in *PTBP1*-KD cells (Figure 2C). Additionally, an increase in the number of cells in the G2/M phase accompanied by a reduction in the G1 phase was observed following *PTBP1* knockdown, suggesting that *PTBP1* silencing could induce cell cycle arrest in the G2/M phase (Figure 2D). Collectively, these outcomes suggest that *PTBP1* is a critical regulator of senescence-associated phenotypes in LUAD cells.

### 3.3. PTBP1 Regulates Expression of Cell Cycle Related Gene FBXO5 Via Alternative Splicing

**Figure 4:**
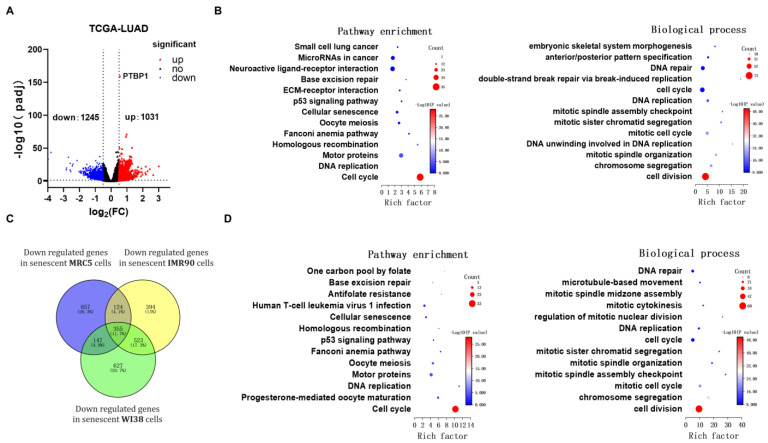
Functional annotation of *PTBP1*-related genes in LUAD and senescence. (**A**) Profile of differentially expressed genes (DEGs) based on the median level of *PTBP1* mRNA, as presented in volcano plots. LUAD patients from the TCGA-LUAD dataset were stratified into *PTBP1*-high and *PTBP1*-low groups. The dotted lines indicates the threshold for |log fold change (FC)| ≥ 0.5 and adjusted *p*-value < 0.05. (**B**) KEGG pathway analysis and Gene Ontology (GO) term enrichment for genes upregulated by *PTBP1* in TCGA-LUAD dataset. (**C**) Venn diagram demonstrating the intersections of downregulated genes across replicative senescence cell lines of three human embryonic lung fibroblasts, MRC5, IMR90, and WI38. (**D**) KEGG pathway analysis and Gene Ontology (GO) term enrichment for overlapping genes across three replicative senescence cell lines.

To study the mechanism by which *PTBP1* induces LUAD cells’ senescence, we first explored *PTBP1*-associated genes in LUAD and senescence based on TCGA and GEO datasets. We examined the differentially expressed genes (DEGs) between *PTBP1*-high and *PTBP1*-low patients in TCGA-LUAD, which were stratified based on the median *PTBP1* mRNA level. We found that 2276 mRNAs (1031 upregulated and 1245 downregulated) were differently expressed in *PTBP1*-high patients compared to *PTBP1*-low ones (Figure 4A). We performed KEGG pathway analysis and GO enrichment analysis on *PTBP1* upregulated genes in the TCGA-LUAD dataset. KEGG pathway analysis revealed that the most enriched genes are associated with the cell cycle and DNA replication. And the most significantly enriched GO terms were related to cell division and chromosome segregation, which aligned with the KEGG enrichment results (Figure 4B). Meanwhile, to further investigate senescence-related pathways, we performed functional enrichment analysis of 355 universally underexpressed genes across replicative senescence cell lines of three types of human embryonic lung fibroblasts: MRC5, IMR90, and WI38 (Figure 4C). KEGG enrichment analysis indicated that the target genes were primarily associated with the cell cycle, and the main terms of the GO analysis involved cell division, which was consistent with the *PTBP1*-related pathway (Figure 4D). This indicated that *PTBP1* might function in cellular senescence by regulating cell-cycle-related genes.

**Figure 5:**
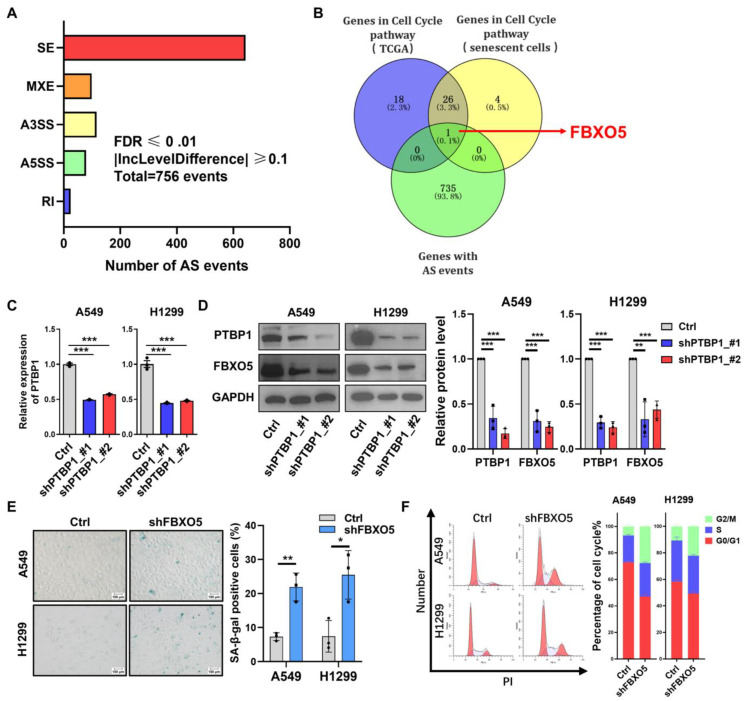
*PTBP1* regulates the expression of cell-cycle-related gene *FBXO5* via alternative splicing. (**A**) Statistics for differential events of alternative splicing following *PTBP1* knockdown in A549 cells (FDR ≤ 0.01, |IncLevelDifference| ≥ 0.1). (**B**) Venn diagram showing overlapping genes between differential differentially spliced genes and cell-cycle-related genes after *PTBP1* knockdown in A549 cells. (**C**) RT-qPCR analysis for mRNA level of *FBXO5* after *PTBP1* depletion in A549 and H1299 cells. (**D**) Western blot analysis of *FBXO5* after *PTBP1* knockdown in A549 and H1299 cells. (**E**) SA-β-Gal staining for A549 and H1299 cells with *FBXO5* knockdown. (**F**) Cell cycle analysis for A549 and H1299 cells with *FBXO5* knockdown. In the graph on the left side, red represents cells in the G1 and G2 phases. (* *p* < 0.05, ** *p* < 0.01, *** *p* < 0.001, *t*-test).

As *PTBP1* is a member of a splicing factor family, the splicing changes in its target genes may help explain *PTBP1*-KD-induced senescence. Accordingly, we next analyzed the alternative splicing patterns affected by *PTBP1* using RNA-seq. We identified 756 alternative splicing events in *PTBP1*-KD A549 cells compared to control ones by analyzing the RNA-seq data with rMATS (Figure 5A). Subsequently, to identify genes related to the cell cycle that are affected by *PTBP1* through its influence on alternative splicing, we took genes from the intersection of alternative splicing events identified in *PTBP1*-KD A549 cells and genes involved in the cell cycle pathway. The green circle represents differential alternative splicing events. The blue and yellow circles represent genes positively correlated with *PTBP1* expression in TCGA-LUAD samples and genes downregulated during replicative senescence in three human embryonic lung fibroblasts (MRC5, IMR90, and WI38) that are involved in the cell cycle pathway from KEGG enrichment. Eventually, *FBXO5* was selected as a candidate (Figure 5B).

**Figure 6:**
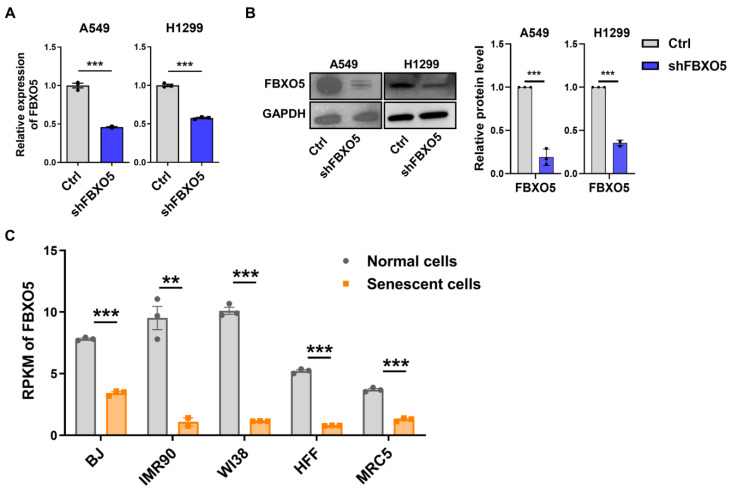
*FBXO5* shows downregulation in senescent cells. *FBXO5* expression was validated in A549 and H1299 cells transfected with sh*FBXO5* by RT-qPCR (**A**) and Western blotting (**B**). GAPDH served as the internal control for both mRNA and protein. (**C**) Decrease in *FBXO5* mRNA in five human senescent models compared to younger ones (** *p* < 0.01, *** *p* < 0.001, *t*-test).

*FBXO5* (F-box protein 5), also known as Early Mitotic Inhibitor 1 (Emi1), plays an essential role in the cell cycle, particularly during mitosis. It is indispensable for the assembly of cyclins and the accumulation of cell cycle regulatory factors during the S and G2 phases [35,36]. *PTBP1* depletion markedly decreased the mRNA and protein expression of *FBXO5* through RT-qPCR and Western blotting in A549 and H1299 cells (Figure 5C,D). Additionally, knockdown of *FBXO5* could induce senescence and G2/M-phase arrest in A549 and H1299 cells (Figure 5E,F and Figure 6A,B). *FBXO5* also displayed decreased expression in five replicative senescence models we previously referenced (Figure 6C). These results suggest that *FBXO5* might be a critical downstream target of *PTBP1* in LUAD.

### 3.4. PTBP1 Mediates Exon 3 Skipping of FBXO5 Pre-mRNA in LUAD

**Figure 7:**
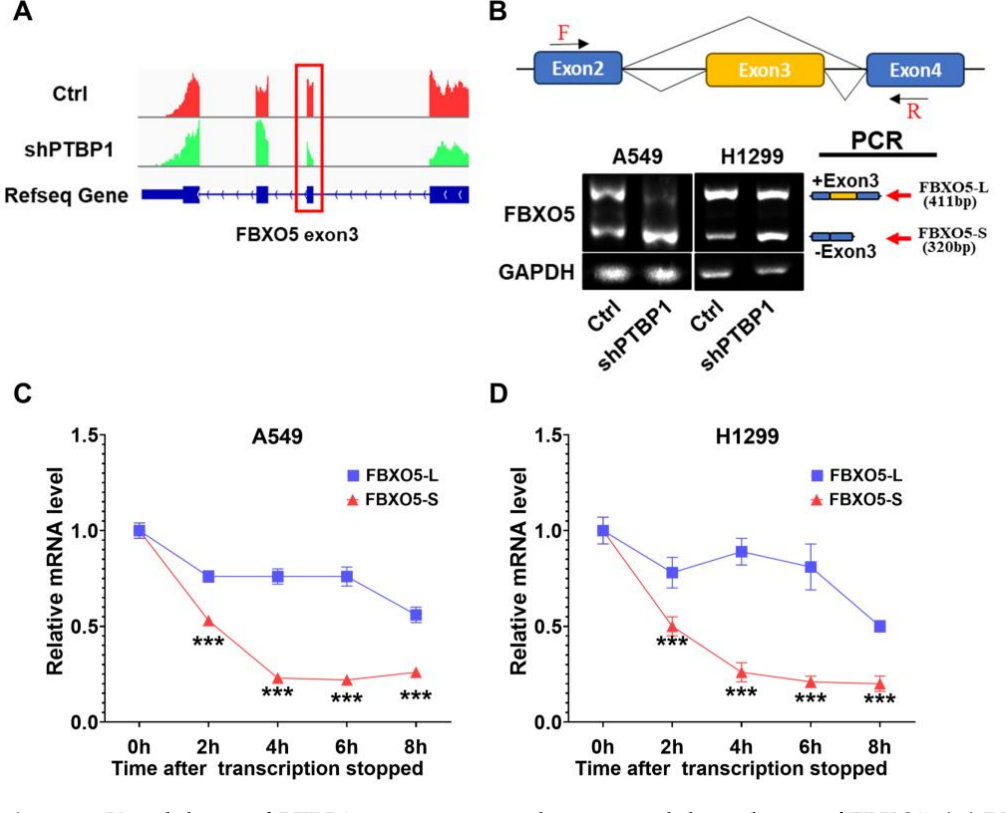
Knockdown of *PTBP1* promotes exon skipping and degradation of *FBXO5*. (**A**) RNA-seq read coverage plots of the gene *FBXO5* in A549 cells after *PTBP1* depletion. Exon numbers and transcript identification numbers of *FBXO5* in the RefSeq annotation are shown. (**B**) RT-PCR was performed to analyze exon 3 skipping of *FBXO5* upon *PTBP1* knockdown in LUAD cells. Primers designed against exon 2 and exon 4 of *FBXO5* are shown above in schematic representation of *FBXO5* splicing variants. Schematic of alternative spliced isoform structures for each PCR product is shown next to the gel image. A higher band intensity of PCR products indicates a higher production of the specific transcript isoform. GAPDH served as the endogenous control. (**C**,**D**) RNA stability assay in A549 (**C**) and H1299 (**D**) cells (*** *p* < 0.001, *t*-test).

After analyzing the alternative splicing pattern of *FBXO5* in the RNA-seq data using the rMATS algorithm, we found that *PTBP1* knockdown promoted exon 3 skipping to generate the short-length isoform of *FBXO5* (FBXO5-S) relative to the full-length isoform of *FBXO5* (FBXO5-L) (Figure 7A). We then validated the alternative splicing pattern of *FBXO5* by RT-PCR using primers designed within exon 2 and exon 4 spanning exon 3 of FBXO5. The results showed that the level of FBXO5-S was increased after *PTBP1* knockdown in A549 and H1299 cells (Figure 7B).

Although, as mentioned above, *PTBP1* depletion markedly decreased the mRNA and protein expression of *FBXO5*, whether the reduced protein level could be attributed to faster RNA degradation or weakened translational efficiency was unclear. To answer this question, we performed an RNA stability assay and found that FBXO5-S had a significantly faster mRNA degradation rate compared to FBXO5-L in A549 and H1299 cells (Figure 7C,D), indicating that RNA stability may explain the reduced mRNA and protein levels of *FBXO5*. These data indicate that *PTBP1* knockdown facilitated splicing of *FBXO5* pre-mRNA into an unstable transcript.

## 4. Discussion

Investigating the tumor-related mechanisms of *PTBP1* is essential, as dysregulation in its expression has been involved in disease promotion, including colorectal cancer invasion, breast and ovarian cancer cell growth, and Parkinson’s disease [37,38,39,40]. However, the influence of *PTBP1* on cellular senescence remains to be elucidated. We found that *PTBP1* exhibited prevalently increased expression in various cancer types and decreased expression in multiple cellular senescence models. Subsequently, we verified that elevated levels of *PTBP1* are commonly observed and associated with a poor prognosis in patients with LUAD. Further research indicated that the depletion of *PTBP1* triggered senescence and hindered LUAD progression.

A growing number of studies have identified that deregulated splicing factors lead to the formation of splice variants, including senescence-associated ones [41,42]. In this study, we revealed that the exon 3 skipping of *FBXO5* was induced by knockdown of *PTBP1* in LUAD cells. *FBXO5* is an endogenous inhibitor of APC/C, which is initially synthesized during the G1-S transition, accumulated in the S and G2 phases, and ultimately degraded by the SCF^β-trcp^ pathway [36,43]. Evidence suggests that *FBXO5* is involved in oncogenesis and the progression of various malignancies [44,45,46]. A study has pointed out that *FBXO5* significantly influences the development and prognosis of lung squamous cell carcinoma (SqCC) [47]. Additionally, cluster statistical analysis revealed that *FBXO5* expression is more pronounced in malignant tumors compared to benign tumors, indicating that there is a significant dysregulation of mitotic APC/C substrates in malignancies, which is absent in benign growths [48]. Two isoforms of *FBXO5*, transcript a and transcript b, have been reported to promote migration and osteogenic differentiation in human periodontal ligament mesenchymal stem cells [27]. However, the relationship between senescence-associated splice variants of *FBXO5* and cellular senescence remains unclear. In our study, we demonstrate that depletion of *PTBP1* induces exon 3 skipping in *FBXO5*, resulting in the generation of the unstable RNA splice variant FBXO5-S and the consequent reduction in *FBXO5* expression. Although, in our study, the regulation of the transition between FBXO5-L and FBXO5-S by *PTBP1* could be considered a cause of cellular senescence, the mechanisms underlying *FBXO5*’s influence on senescence warrant further investigation.

Cellular senescence, a critical process in aging and disease, is marked by indicators such as SA-β-Gal, SASP, P16, and P21 [49]. In our research, we found that knocking down *PTBP1* increases SA-β-Gal staining, which is considered the gold standard for cellular aging. Intriguingly, *PTBP1* has been identified as a regulator of alternative splicing for genes implicated in intracellular trafficking, including *EXOC7*, which is instrumental in controlling SASP expression. Targeting *PTBP1* for inhibition is linked to a decreased risk of tumorigenesis and the attenuation of SASP’s pro-tumorigenic properties [23]. SASP factors are known for their dual role in both promoting and inhibiting tumorigenesis, depending on the context [7]. Consequently, the silencing of *PTBP1* may induce cellular senescence and suppress the release of tumor-associated SASP, indicating that the repression of *PTBP1* may exert an anti-tumorigenic effect by promoting senescence while deterring its cancer-promoting potential. Future studies will evaluate the potential role of *PTBP1* in the influence of the tumor-promoting aspects of SASP in LUAD.

## Figures and Tables

**Table 1:** Details of shRNA sequences used for cell transfection.

Target	Primer Sequence
shPTBP1_#1	5′-CTCAACGTCAAGTACAACAAT-3′
shPTBP1_#2	5′-AGCAAACGGAAATGACAGCAA-3′
shFBXO5	5′-CCAGACCAATATCCCAACAAA-3′

**Table 2:** PCR primer lists.

Target	Primer Sequence
RT-qPCR	
GAPDH	Forward: CTGGGCTACACTGAGCACC
	Reverse: AAGTGGTCGTTGAGGGCAATG
PTBP1	Forward: AGCGCGTGAAGATCCTGTTC
	Reverse: CAGGGGTGAGTTGCCGTAG
FBXO5	Forward: CAGCGAACTCTTTCGAAGGGGACTC
	Reverse: GTGAATTACAGCGAATACAGGCTTTGAGGC
FBXO5-S	Forward: GCACAACTCAGTGACATGGACTTAATCAAGAAAAC
	Reverse: CTGCTGATTTCTGAACAGAAGCCAGTGG
FBXO5-L	Forward: CCAGTTGTACAGTAAAGCAATACAAAGAGTTACCGAAAAC
	Reverse: CTGCTGATTTCTGAACAGAAGCCAGTGG
RT-PCR	
GAPDH	Forward: CTGGGCTACACTGAGCACC
	Reverse: AAGTGGTCGTTGAGGGCAATG
FBXO5exon 2-exon 4	Forward: CAGCGAACTCTTTCGAAGGGGACTC
	Reverse: GGTGAATTACAGCGAATACAGGCTTTGAGGC

## Data Availability

The GSE datasets were obtained from the GEO profiles section at https://www.ncbi.nlm.nih.gov (accessed on 10 September 2023).
